# Clinical significance of mitofusin-2 and its signaling pathways in hepatocellular carcinoma

**DOI:** 10.1186/s12957-016-0922-5

**Published:** 2016-07-07

**Authors:** Yingsheng Wu, Dongkai Zhou, Xiaobo Xu, Xinyi Zhao, Pengfei Huang, Xiaohu Zhou, Wei Song, Hua Guo, Weilin Wang, Shusen Zheng

**Affiliations:** Division of Hepatobiliary and Pancreatic Surgery, Department of Surgery, First Affiliated Hospital, School of Medicine, Zhejiang University, No. 79 Qingchun Road, Hangzhou, 310003 China; Key Lab of Combined Multi-organ Transplantation, Ministry of Public Health, No. 79 Qingchun Road, Hangzhou, 310003 China; Collaborative Innovation Center for Diagnosis and Treatment of Infectious Diseases, No. 79 Qingchun Road, Hangzhou, 310003 China

**Keywords:** MFN2, Hepatocellular carcinoma, Overall survival, Network function

## Abstract

**Background:**

The mitochondrial GTPase mitofusin-2 (*MFN2*) gene encodes a mitochondrial membrane protein that can induce apoptosis of hepatocellular carcinoma (HCC) via the mitochondrial apoptotic pathway, as validated in our previous research. However, little is known of the clinical significance of MFN2 expression and its signaling pathways in HCC.

**Methods:**

MFN2 mRNA expression in tumor and adjacent non-tumor tissues from 115 patients with HCC was investigated using quantitative real-time PCR. The association of the MFN2 mRNA expression level with clinical and pathological parameters was evaluated statistically, while a comparative microarray analysis was used to identify MFN2 signaling pathways in HepG2 cells.

**Results:**

MFN2 was significantly (*p* < 0.0001) downregulated in HCC tissues. Low MFN2 expression was significantly correlated with sex and preoperative alpha-fetoprotein (*p* < 0.05). Both a Kaplan–Meier survival curve and multivariate analyses showed that MFN2 was related to overall survival. A comparative gene expression microarray revealed 211 upregulated (58 %) and 153 downregulated (42 %) genes. Eighteen pathways were identified as the most significant pathways correlated with MFN2.

**Conclusions:**

Low MFN2 expression in HCC indicated a worse overall survival. Crucial signaling molecules such as PI3K-AKT, cytokine receptor, and focal adhesion may participate in MFN2-mediated signaling pathway changes in HCC.

**Electronic supplementary material:**

The online version of this article (doi:10.1186/s12957-016-0922-5) contains supplementary material, which is available to authorized users.

## Background

Hepatocellular carcinoma (HCC) is one of the most lethal cancers [1], accounting for approximately 600,000 deaths annually worldwide [2]. Viral infections, excessive alcohol intake, and exposure to aflatoxin are common risk factors for developing HCC. In China and Africa, hepatitis B virus causes more than 50 % of HCC, whereas hepatitis C virus is the leading cause in Europe and North America [3]. In addition, hereditary liver diseases and non-alcoholic fatty liver disease are associated with HCC. The pathogenesis of HCC includes the stepwise development of liver injury, regeneration, fibrosis and cirrhosis, dysplasia, and malignancy, which ultimately transforms normal liver cells into cancer cells via genetic and epigenetic alterations [4]. To comprehend the molecular biology of HCC, recent whole-genome research and exome sequencing analyses have found key pathway changes in HCC, including inactivation of the p53 pathway and activation of the wnt/β-catenin and Ras/PI3K pathways and telomerase [5].

The mitofusin 2 (*MFN2*) gene, also known as the hyperplasia suppressor gene, encodes a protein belonging to the GTPase family that is located on the mitochondrial outer membrane [6, 7] and is involved not only in mitochondrial fusion but also in mitochondrial trafficking and mitophagy [8]. Mutations or abnormalities of MFN2 may occur in various diseases, including Charcot–Marie–Tooth disease, obesity, and diabetes mellitus [9–11]. Recent studies have shown that MFN2 overexpression suppresses the proliferation of vascular smooth muscle cells and cardiac myocytes in the rat [12, 13]. Our research team has reported on the pro-apoptosis effects of MFN2 in HCC over the past 5 years [14–16]. Furthermore, MFN2 acts as a tumor suppressor in diverse cancers of the bladder, stomach, and lung [17–19].

However, the relationship between MFN2 expression and the clinical characteristics of HCC has not been explored. Therefore, we investigated MFN2 mRNA expression in tumor and adjacent non-tumor tissues from 115 patients with HCC and statistically evaluated the association of MFN2 mRNA expression with clinical and pathological parameters. A gene expression microarray was used to determine whether MFN2 correlated with differentially expressed genes (DEGs) in HepG2 cells. We also constructed an MFN2-related functional interaction (FI) network by mapping these DEGs to the FI data.

## Methods

### Patients

This study enrolled 115 patients with HCC who underwent curative hepatectomy. The study was approved by the ethics committee of the First Affiliated Hospital of Zhejiang University, and informed consent was obtained from all patients. HCC was diagnosed in all patients before or after hepatectomy and was confirmed histopathologically. None of the patients received presurgical chemotherapy or radiation therapy. The baseline characteristics of the patients are summarized in Additional file 1: Table S1. Most patients were followed regularly as outpatients. A diagnosis of recurrence was based on typical contrast computed tomography (CT) or magnetic resonance imaging (MRI) findings.

### Cell lines and cell culture

The HepG2 HCC cell line was cultured in Dulbecco’s modified Eagle’s medium (DMEM) (Gibco, Grand Island, NY, USA) supplemented with 10 % heat-inactivated fetal bovine serum (Sigma-Aldrich, St. Louis, MO, USA) and 100 U/mL penicillin/streptomycin. HepG2 cells were maintained in a humidified atmosphere containing 5 % CO_2_ at 37 °C and were passaged using standard cell culture techniques.

### Total RNA extraction and cDNA synthesis

Total RNA was extracted using TRIzol reagent (Invitrogen, Carlsbad, CA, USA), according to the manufacturer’s protocol. The concentration and purity of RNA were assessed spectrophotometrically at 260 and 280 nm. cDNA was synthesized from total RNA (2 μg) using M-MLV Reverse Transcriptase (Promega, San Luis Obispo, CA, USA), following the manufacturer’s instructions.

### Quantitative real-time PCR

Quantitative real-time PCR was performed with the ABI PRISM 7500 Sequence Detection System (Applied Biosystems) using a SYBR Premix DimerEraser kit (Takara Biotechnology, Dalian, Liaoning, China). Amplification reactions included 1 μl cDNA template, 0.3 μl each of the forward and reverse primers (10 μM), 0.2 μl of 50× ROX Reference Dye II (Takara), and 5 μl of 2× SYBR Premix DimerEraser in a total volume of 10 μl. The primers used were 5′-AATCTGAGGCGACTGGTGA-3′ (forward) and 5′-CTCCTCCTGTTCGACAGTCA-3′ (reverse) for MFN2 and 5′-CTTAGTTGCGTTACACCCTTTC-3′ (forward) and 5′-CACCTTCACCGTTCCAGTTT-3′ (reverse) for β-actin. The transcripts were amplified with an initial denaturation at 95 °C for 30 s, followed by 40 cycles at 95 °C for 5 s, 55 °C for 30 s, and 72 °C for 34 s. The comparative threshold cycle (2^−ΔΔCT^) method was used for relative quantification. β-Actin was used as an internal control for normalization. All real-time PCRs were performed in triplicate to evaluate data reproducibility.

### Plasmid transfection

The plasmid vector (pIRES2-EGFP Vector) and plasmid-MFN2 were purchased from Invitrogen, USA. The plasmids were transfected in HepG2 cells using Lipofectamine 2000 (Invitrogen), according to the manufacturer’s instructions. The efficiency of transfection was evaluated by qPCR and western blot analysis after plasmid treatment for 48 h.

### Gene expression microarray

Total RNA was extracted from HepG2 cells transfected with plasmid vector-NC (pIRES2-EGFPVector) or plasmid-MFN2 for 48 h using TRIzol (Invitrogen). The Affymetrix Human Genome U133Plus 2.0 Array (Affymetrix, USA) was used for gene expression profiling. Microarray experiments were performed at ShanghaiBio (National Engineering Center for Biochips, Shanghai, China). MAS 5.0 and the “Oligo” package from Bioconductor (http://www.bioconductor.org) were used to normalize the data and annotate probe information.

### Data preprocessing and identification of DEGs

Normalized signal intensity data were imported into BRB-ArrayTools ver. 4.5 (National Cancer Institute, http://linus.nci.nih.gov/BRB-ArrayTools.html) for preprocessing. We excluded those genes for which the percentage absent exceeded 50 %. We identified DEGs using paired *t* tests with a random variance model. The nominal significance level for each univariate test was <0.05. Only genes with a fold change of ≥2 were selected as DEGs.

### Construction of the MFN2-related FI network

In total, 217,249 pairs of FIs were downloaded from Reactome [20] (ver. 2014, http://www.reactome.org). These pairwise relationships are derived from datasets of protein–protein interactions from BioGRID [21], the Database of Interacting Proteins [22], the Human Protein Reference Database [23], I2D [24], IntACT [25], and MINT [26]. This interaction information was imported into Cytoscape ver. 3.2.1 [27] (http://www.cytoscape.org). By mapping the MFN2-related DEGs to the FI data, we constructed the MFN2-related FI network.

### Pathway enrichment analysis for the FI network

The ReactomeFIViz app was used in Cytoscape for pathway enrichment analysis [28]. The sources of pathway annotations include Cell Map (http://cancer.cellmap.org), Reactome [20], the Kyoto Encyclopedia of Genes and Genomes (KEGG) [29], Panther Pathways [30], NCI-PID [31], and BioCarta (http://www.biocarta.com/genes/index.asp). A false discovery rate (FDR) of <0.05 was selected as the cutoff criterion.

### Statistical analysis

Statistical analyses of continuous variables are presented the mean ± standard deviation. The non-parametric Mann–Whitney *U* test was used to analyze differences in the MFN2 mRNA expression between the tumor tissue and corresponding non-tumor tissue. Pearson’s chi-square test was used to compare categorical variables, whereas Student’s *t* test was used for continuous variables. We divided the patients into high and low expression groups using the median MFN2 mRNA expression as a cutoff because the median is not affected by extreme values (outliers). Survival curves were generated using the Kaplan–Meier method, and the differences were compared using the log-rank test. Multivariate analysis was performed using the Cox proportional hazard regression model. A two-tailed *p* value of <0.05 was considered statistically significant. All statistical analyses were performed using the Statistical Package for the Social Sciences (SPSS 20.0 for Windows, SPSS, Chicago, IL). Graphs were created using GraphPad Prism (ver. 6.01).

## Results

### Expression of MFN2 mRNA in HCC paired tissues

MFN2 mRNA levels were determined in 115 pairs of human HCC and corresponding non-tumor hepatic tissues. The MFN2 mRNA expression was higher in 76.5 % of the non-tumor hepatic tissues than in the paired HCC tissue (Additional file 2: Figure S1A). MFN2 was significantly (*p* < 0.0001) downregulated in tumor tissue compared with non-tumor tissue (Additional file 2: Figure S1B), with average mRNA expression levels of 6.76 ± 8.04 and 4.34 ± 6.06, respectively (Table 1).

**Table 1:** Distribution of *MFN2* mRNA level and survival in HCC patients

Variables	Mean ± SD	Range	Percentiles		
			25	50	75
*MFN2* in non-tumor tissues	6.76 ± 8.04	0.32–58.06	1.55	4.05	9.32
*MFN2* in tumor tissues	4.34 ± 6.06	0.20–30.36	0.99	1.81	4.66

*MFN2* mitofusion 2

### Correlation between MFN2 expression and clinicopathological parameters

The patients with HCC were divided into low (*n* = 58) and high (*n* = 57) expression groups based on the median value of MFN2 expression. Table 2 lists the patients’ clinicopathological variables. Sex and preoperative alpha-fetoprotein correlated significantly (*p <* 0.05) with MFN2 mRNA expression, whereas age, HBsAg, HBV-DNA replication, liver cirrhosis, tumor number, tumor size, vascular invasion, lymph node metastasis, intrahepatic metastasis, liver capsular invasion, differentiation, and TNM stage did not.

**Table 2:** Correlation between *MFN2* expression in tumor tissue with clinicopathological factors in hepatocellular carcinoma patients

Variables	*MFN2* expression		
	Low	High	*P* value
	*n* = 58	*n* = 57	
Age (year)	57.1 ± 11.4	57.1 ± 9.9	0.996
Gender (female/male)	3/55	12/45	0.012
HBsAg (no/yes)	15/43	7/50	0.065
HBV-DNA replication (no/yes)	34/24	32/25	0.789
Liver cirrhosis (no/yes)	22/36	18/39	0.476
Preoperative AFP	6292.3 ± 14073.3	4075.1 ± 13793.9	0.048
Tumor number (1/>1)	44/14	42/15	0.789
Tumor size (cm)	6.9 ± 3.6	6.3 ± 3.0	0.610
PV or VI invasion (no/yes)	37/21	43/14	0.177
Lymph node metastasis (no/yes)	51/7	47/10	0.410
Intrahepatic metastasis (no/yes)	38/20	33/24	0.402
Liver capsular invasion (no/yes)	40/18	41/16	0.729
TNM stage (I/II–IV)	23/35	22/35	0.908
Differentiation (well/moderate or poor)	20/38	28/29	0.113

*HBsAg* hepatitis B surface antigen, *HBV-DNA* hepatitis B virus deoxyribonucleic acid, *AFP* alpha-fetoprotein, *PV* portal vein, *VI* intrahepatic vein

### Prognostic significance of MFN2 expression

**Fig. 1:**
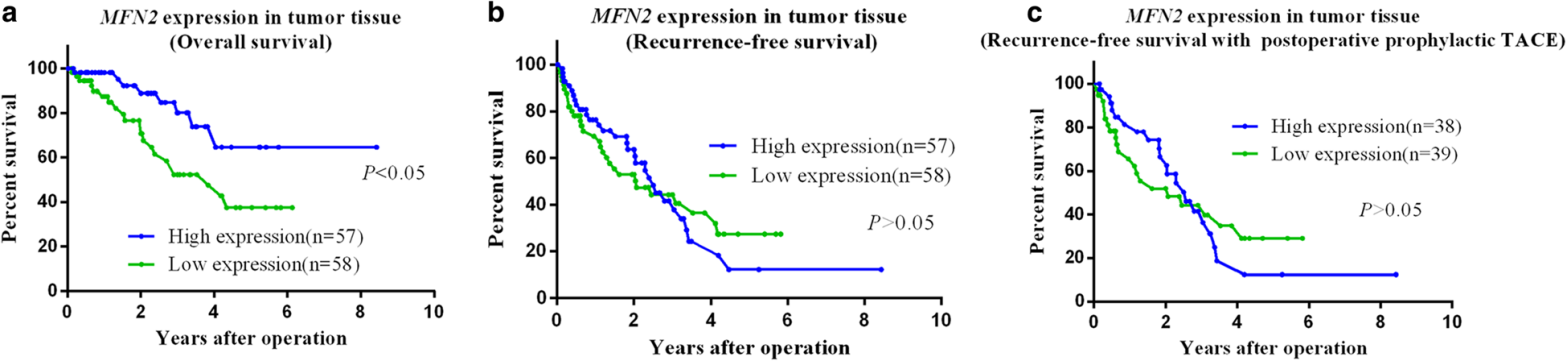
Survival curves for patients with HCC with high and low MFN2 expression were plotted using the Kaplan–Meier method, and the differences were evaluated using the log-rank test. **a** MFN2 expression differed significantly with the overall survival rates between the two groups. **b**, **c** However, no significant difference was found in the recurrence-free survival rates, even considering postoperative prophylactic TACE

Evaluating the relationship between MFN2 mRNA expression and patient survival using the Kaplan–Meier survival curve, patients with relatively high MFN2 expression in HCC had substantially longer overall survival (OS) than those with low MFN2 expression (*p <* 0.05) (Fig. 1a). However, MFN2 expression did not seem to affect the recurrence-free survival (*p >* 0.05) (Fig. 1b), even after considering postoperative prophylactic transhepatic arterial chemotherapy and embolization (TACE) (Fig. 1c).

Furthermore, univariate analysis revealed that liver capsule invasion and MFN2 expression were significant predictors of OS (Table 3). Multivariate analysis using the Cox proportional hazards model also indicated that liver capsule invasion (hazard ratio (HR) = 7.206, *p =* 0.011) and MFN2 expression (HR = 0.063, *p =* 0.009) were independent predictors in patients with HCC (Table 3).

**Table 3:** Risk factor analysis of overall survival in tumor tissue

Prognositic factors	Univariate analysis		Multivariate analysis	
	HR (95 % CI)	*P* value	HR (95 % CI)	*P* value
Age (<60/≥60)	0.704 (0.250–1.983)	0.507		
Gender (female/male)	0.263 (0.034–2.007)	0.197		
HBsAg (no/yes)	1.688 (0.379–7.521)	0.492		
HBV-DNA replication(no/yes)	1.491 (0.539–4.122)	0.441		
Liver cirrhosis (no/yes)	1.117 (0.355–3.515)	0.850		
Preoperative AFP (<20/≥20 ng/ml)	0.741 (0.262–2.100)	0.573		
Preoperative AFP (<400/≥400 ng/ml)	1.033 (0.351–3.035)	0.954		
Tumor number (1/>1)	0.861 (0.251–3.174)	0.893		
Tumor size (<5/≥5 cm)	1.304 (0.462–3.677)	0.616		
Tumor size (<8/≥8 cm)	2.193 (0.729–6.594)	0.162		
PV or VI invasion (no/yes)	0.959 (0.268–3.433)	0.949		
PVTT (no/yes)	0.517 (0.067–3.973)	0.526		
Lymph node metastasis (no/yes)	0.773 (0.098–6.077)	0.807		
Intrahepatic metastasis (no/yes)	1.264 (0.429–3.726)	0.670		
Liver capsular invasion (no/yes)	5.811 (1.975–17.096)	0.001	7.206 (1.571–33.063)	0.011
TNM stage (I/II–IV)	2.322 (0.772–6.980)	0.134		
Differentiation (well/moderate or poor)	1.529 (0.541–4.318)	0.423		
TACE (no/yes)	0.877 (0.276–2.794)	0.825		
*MFN2* expression (low/high)	0.263 (0.074–0.933)	0.039	0.063 (0.008–0.496)	0.009

*HBsAg* hepatitis B surface antigen, *HBV-DNA* hepatitis B virus deoxyribonucleic acid, *AFP* alpha-fetoprotein, *PV* portal vein, *VI* intrahepatic vein, *PVTT* portal vein tumor thrombus, *MFN2* mitofusion 2, *TACE* transcatheter arterial chemoembolization

### Identification of DEGs after MFN2 overexpression

In total, 364 genes were differentially expressed (Additional file 3: Table S2) in HepG2 cells transfected with plasmid-MFN2 compared with HepG2 cells transfected with vector-NC (Additional file 5: Figure S2). Of these, 211 genes (58 %) were upregulated and the remaining 153 (42 %) were downregulated.

### Analysis of FI network affected by MFN2

**Fig. 2:**
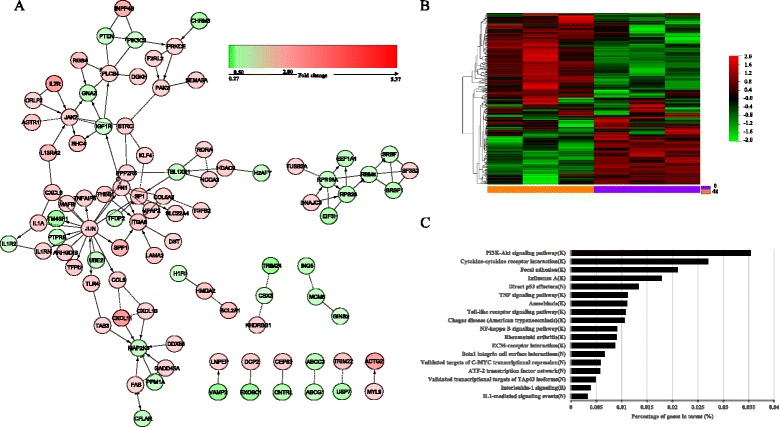
**a** Functional interaction (FI) network constructed using MFN2-related differentially expressed genes. Edges are displayed based on FI annotation, including “->” for activating/catalyzing, “-|” for inhibition, “-” for FIs extracted from complexes or inputs, and “---” for predicted FIs. Node colors represent the fold changes in MFN2-related DEGs, ranging from *red* for high expression to *green* for low expression, relative to vector-NC. **b** Heat map of the 93 MFN2-related differentially expressed genes. **c** The pathway analyses of differentially expressed genes identified by microarray

By mapping the MFN2-related DEGs to the FI data, we constructed the MFN2-related FI network. This network comprised 93 nodes in 11 clusters, with the largest cluster containing 62 nodes (Fig. 2a). Using hierarchical clustering based on the gene expression level (Fig. 2b), the 93 genes in the network could be differentiated into two groups. The 93 nodes were connected via 114 FIs, corresponding to an effective mean degree of 2.5. Degree refers to the number of nearest neighbors of a node. Nodes with degrees of ≥5 were selected as hub nodes. The hub nodes in the FI network were JUN, JAK2, FN1, MAP2K6, ITGA6, RPS15A, PLCB4, RBM8A, RPS28, IGF1R, TBL1XR1, and SP1, suggesting that these genes are related to MFN2.

To functionally classify these 93 significant genes, we used ReactomeFIViz to identify significant enrichment of these genes in 18 pathways (Fig. 2c, Additional file 4: Table S3). The most significant pathways were the PI3K–Akt signaling pathway, cytokine–cytokine receptor interaction, focal adhesion, influenza A, and direct p53 effectors.

## Discussion

Mitofusin 2 was first recognized as a key protein not only regulating mitochondria fusion but also participating in tumor cell proliferation. Although MFN2 has not been proven to act as a tumor suppressor gene in cancer cells [32], its antitumor function, as revealed in various tumors, continues to be accepted. Previously, we proved that overexpression of the *MFN2* gene in HCC resulted in tumor cell apoptosis via mitochondrial pathways mediated by calcium influx [14–16]. MFN2 is a downstream target gene of P53 [33], and such direct regulation is altered by hepatitis B virus X protein in HCC [34]. Here, we examined the clinical significance of MFN2 mRNA expression in 115 HCC specimens. MFN2 was downregulated dramatically in HCC tissues, which is consistent with our previous findings. In fact, we have investigated the possible mechanism of MFN2 downregulation for about 8 years. Firstly, we suspected MFN2 gene downregulated by aberrant promoter CpG methylation. However, we found normal methylation level in MFN2 gene promoter region (data not shown here). Secondly, a lot of potential upstream genes of MFN2 were chosen to be investigated. Fortunately, we found that MFN2 is a novel target of P53 which may partly explain the low expression level of MFN2 in HCC [33]. Furthermore, MicroRNAs were considered as candidate regulation factors of MFN2. And we did demonstrate an upregulated MicroRNA called miR761 that could directly regulate MFN2 in HCC [35]. We suggest that the possible molecular mechanism behind MFN2 downregulated is very complicated that cannot be clarified by one factor recently. Maybe, a new critical factor served as a MFN2 regulator will be found in the future.

According to statistical analysis in Table 2, we found that tumor MFN2 expression was significantly correlated with sex and the preoperative alpha-fetoprotein level. A recent paper reported significant differences in the expression of a key regulator of mitochondrial biogenesis between males and females in the mouse brain [36]. Therefore, it is possible that MFN2 is expressed differently in human males and females. However, there may be sampling errors in these studies; a larger sample is needed to confirm this finding. Additionally, as a tumor marker, AFP served as an important indicator for HCC diagnosis and patient follow-up. In our findings, preoperative AFP level in serum and MFN2 mRNA level in HCC tissue showed opposite tendency, hypothesized a potential regulation relationships between them. However, we had not found any evidence to demonstrate this hypothesis yet. Combined with survival analysis, we thought the patient who had a high level of MFN2 and low level preoperative AFP may have a better overall survival after operation. But such interpretation was not logical because various factors could affect overall survival rate after hepatectomy for HCC patient. Perhaps, it is better to correlate MFN2 with postoperative AFP. However, we were unable to obtain all postoperative AFP level in this study.

Despite the weak relationships between MFN2 expression and clinicopathological parameters, survival analysis showed that a higher MFN2 expression level was associated with better postoperative survival of patients with HCC. Furthermore, univariate analysis revealed that liver capsule invasion and MFN2 expression were significant predictors of OS. Therefore, MFN2 has an important role in the development of HCC. We propose that MFN2 could serve as a biomarker in HCC tissue for predicting survival after hepatectomy.

Most research of MFN2 in tumors has concentrated on the regulation of mitochondrial function. For a more comprehensive evaluation of MFN2-associated genes, we used a comparative gene expression microarray. This microarray revealed 364 DEGs after MFN2 overexpression, of which 93 significant genes were classified functionally into 18 pathways, including the PI3K–Akt signaling pathway, cytokine–cytokine receptor interaction, focal adhesion, and direct p53 effectors. As we know, these pathways play important roles in tumor development and progression [37–40]. Multiple genes, such as mTOR, NF-kB, BCL2, and BAX, participate in PI3K-AKT signal pathway in HCC [41–43]. Therefore, MFN2 may function powerfully through regulating PI3K-AKT. For cytokine–cytokine receptor interaction and focal adhesion, it makes MFN2 possible to regulate such membrane receptors result in tumor cell migration and invasion which will bring us new sight about how MFN2 inhibits tumor cell metastasis. Previous research has found that MFN2 was related to virus infection mechanism [44] which could verify our findings with respect to influenza A. It is interesting that the relationship between MFN2 and P53 may create a positive feedback mechanism based on our findings [33]. These results support previous studies of MFN2 in HCC and may shed new light on the complicated proapoptotic and antiproliferative mechanism of MFN2 in the tumor.

In the future, our first-step study on MFN2 may focus on verifying the comparative microarray analysis results by various experimental methods. After that, we will select a promising downstream pathway of MFN2 to further investigate the regulation mechanism. A cell membrane receptor regulated by MFN2 is also taken into our account in our future research.

## Conclusions

In conclusion, our data suggest that the MFN2 expression level in tumors is closely related to the survival of patients with HCC after hepatectomy. Numerous critical signaling pathways take part in the MFN2-mediated functional changes in HCC.

## Abbreviations

CT, computed tomography; DEGs, differentially expressed genes; DMEM, Dulbecco’s modified Eagle’s medium; FI, functional interaction; HCC, hepatocellular carcinoma; KEGG, Kyoto Encyclopedia of Genes and Genomes; MFN2, mitofusin-2; MRI, magnetic resonance imaging; OS, overall survival

## Additional files

Additional file 1: Table S1: Patients demographic and clinicopathological characteristics. (DOC 52 kb) Additional file 2: Figure S1: Expression of MFN2 mRNA in HCC tumors and corresponding non-tumor hepatic tissues. (A) MFN2 was downregulated in most HCC tumors (76.5 %). (B) MFN2 was significantly (*p* < 0.0001) downregulated in the tumor compared with the non-tumor hepatic counterpart using the non-parametric Mann–Whitney *U* test. Standard error of the mean (SEM) was used as error bar. (TIF 59 kb) Additional file 3: Table S2: DEGs after MFN2 overexpression in HepG2 cells. (DOC 197 kb) Additional file 4: Table S3: Significant enrichment of 93 genes in 18 pathways. (DOC 47 kb) Additional file 5: Figure S2: Efficiency of transfection evaluation by qPCR and western blot analysis in HepG2 cells. (A) MFN2 mRNA was significantly upregulated by plasmid-MFN2. Standard deviation (SD) was used as error bar. (B) MFN2 protein was also upregulated by plasmid-MFN2. (TIF 46 kb)
