# Supplementary material for: Clinical significance of mitofusin-2 and its signaling pathways in hepatocellular carcinoma
Source: World J Surg Oncol. 2016 Jul 7;14:179. doi: 10.1186/s12957-016-0922-5 (PMC4936233; doi:10.1186/s12957-016-0922-5)
Supplement: Additional file 1: Table S1: — Patients demographic and clinicopathological characteristics. (DOC 52 kb) [file 12957_2016_922_MOESM1_ESM.doc]

Supplementary Table 1 Patients demographic and clinicopathological characteristics

| Characteristics | Enrolled patients (n = 119) |
| --- | --- |
| Age  (<60/>60) | 63/52 |
| Gender  (Female/Male) | 15/100 |
| Hepatitis B virus surface antigen  (no/yes) | 22/93 |
| Hepatitis B virus deoxyribonucleic acid replication |  |
| (no/yes) | 66/49 |
| Liver cirrhosis |  |
| (no/yes) | 40/75 |
| Alpha fetoprotein  (<20/>20ng/ml) | 47/68 |
| Alpha fetoprotein  (<400/>400ng/ml) | 69/46 |
| Tumor number  (Single/Multiple) | 86/29 |
| Average tumor size  (<5/>5cm) | 44/71 |
| Average tumor size  (<8/>8cm) | 78/37 |
| Portal vein tumor thrombus |  |
| (no/yes) | 92/23 |
| Portal vein and/or intrahepatic vein invasion  (no/yes) | 80/35 |
| Lymph node metastasis  (no/yes) | 98/17 |
| Intrahepatic metastasis  (no/yes) | 71/44 |
| Liver capsular invasion |  |
| (no/yes) | 81/34 |
| Tumor recurrence  (no/yes) | 55/60 |
| Tumor differentiation  (well/ moderate/poor)  TNM stage | 5/45/65 |
| (Ⅰ/Ⅱ-Ⅳ) | 45/70 |
| Trans-catheter arterial chemoembolization |  |
| (no/yes) | 38/77 |
