# Supplementary material for: Clinical significance of mitofusin-2 and its signaling pathways in hepatocellular carcinoma
Source: World J Surg Oncol. 2016 Jul 7;14:179. doi: 10.1186/s12957-016-0922-5 (PMC4936233; doi:10.1186/s12957-016-0922-5)
Supplement: Additional file 3: Table S2: — DEGs after MFN2 overexpression in HepG2 cells. (DOC 197 kb) [file 12957_2016_922_MOESM3_ESM.doc]

Supplementary Table 2 DEGs after MFN2 overexpression in HepG2 cells

| Symbol | Fold-change | Symbol | Fold-change | Symbol | Fold-change |
| --- | --- | --- | --- | --- | --- |
| MFN2 | 80.72 | ADAMTS5 | 0.42 | GINS2 | 0.48 |
| SUZ12P1 | 0.4 | AFF4 | 2.18 | LRCH3 | 0.49 |
| SRSF1 | 0.41 | GBP1 | 2.78 | DIEXF | 0.49 |
| FLJ16734 | 0.31 | CLN8 | 2.25 | ZNF79 | 2.04 |
| LY6K | 0.47 | SPP1 | 3.75 | WDFY2 | 2.08 |
| NUDT3 | 0.36 | TBL1XR1 | 0.45 | CCL26 | 2.55 |
| INTU | 2.44 | BACE2 | 0.49 | ALDH5A1 | 0.48 |
| CDCA7 | 0.5 | SDCBP2-AS1 | 0.29 | GLS | 2.43 |
| STRN | 0.38 | IL7R | 5.15 | MID1 | 0.35 |
| FAS | 2 | SORBS2 | 0.3 | MFSD9 | 2.86 |
| SAMD9 | 2.01 | RPS15A | 0.47 | MCM6 | 0.44 |
| NACC2 | 2.23 | NUDT3 | 0.42 | RPS28 | 0.43 |
| PLSCR4 | 2.22 | TMEM52B | 2.48 | CDC14B | 2.31 |
| AFF4 | 2.24 | TOR1AIP2 | 2.35 | LGALS8 | 2.21 |
| IFI44 | 2.23 | FLJ37453 | 0.45 | FBLN7 | 0.35 |
| SRPX2 | 2.36 | ASNS | 0.42 | LINC00894 | 0.43 |
| TPK1 | 0.49 | DENR | 3.37 | STYK1 | 2.53 |
| GPR87 | 3.28 | SPRED1 | 2.82 | USH1C | 0.37 |
| MACROD1 | 0.37 | GPRIN3 | 0.37 | ACTR2 | 2.06 |
| LINC00621 | 0.48 | DDX58 | 2.18 | GBP1 | 2.44 |
| DIO2 | 2.3 | SESN2 | 2.43 | FN1 | 2.55 |
| GLIPR1 | 2.47 | REEP3 | 2.18 | GPRC5B | 2.53 |
| AIM2 | 2.04 | KIAA0430 | 0.49 | NUDT3 | 0.35 |
| SESN2 | 3.04 | SMOC1 | 0.44 | UHMK1 | 2.09 |
| Symbol | Fold-change | Symbol | Fold-change | Symbol | Fold-change |
| TMEM237 | 0.48 | BTRC | 2.4 | FAM210B | 0.48 |
| EIF4EBP2 | 0.43 | GBP1 | 2.67 | CDC42EP3 | 2.28 |
| SERPINB5 | 2.29 | PDE12 | 2.11 | RORA | 2.13 |
| GAS1 | 0.5 | CTSF | 0.48 | DYX1C1 | 2.02 |
| KMO | 4.07 | GNAZ | 0.44 | H1FX | 0.45 |
| RSPO3 | 2.14 | PTEN | 0.46 | GABPB1-AS1 | 0.43 |
| BMP2K | 2.46 | IQCE | 2.67 | WAC | 0.49 |
| DCP2 | 2.46 | YKT6 | 2.13 | MRPS14 | 0.46 |
| SPINK1 | 2.09 | F2RL2 | 2.07 | DNAJC3 | 2.22 |
| NCOA3 | 2.23 | ACTG2 | 4.86 | LOC100291323 | 0.49 |
| ZNF37BP | 0.42 | UHMK1 | 2.02 | C12ORF5 | 2.02 |
| EID1 | 0.46 | SLC22A4 | 2.06 | WFS1 | 2.3 |
| LINC00165 | 0.46 | LOC101927809 | 0.26 | IGF1R | 0.49 |
| LINC00963 | 0.46 | IGDCC4 | 3.91 | SP1 | 2.67 |
| CXCL11 | 2.85 | SLC4A7 | 2.08 | PTHLH | 2.13 |
| MMAB | 0.39 | SETX | 2.27 | LOC100507557 | 0.41 |
| SEPP1 | 0.35 | TMEM185B | 0.48 | GAN | 2.66 |
| ABHD2 | 2.11 | GLIPR1 | 2.23 | SH3YL1 | 0.31 |
| PAQR6 | 0.47 | GLIPR1 | 2.34 | RNF182 | 0.49 |
| AREG | 2.11 | UBE2I | 0.47 | SHC4 | 2.36 |
| ATG2B | 0.45 | TGM2 | 2.02 | TUBB2A | 2.26 |
| AKR1B10 | 2.61 | EIF4EBP2 | 0.47 | ANKRD9 | 0.47 |
| ARL14 | 2.87 | TFDP2 | 0.49 | ETV2 | 0.49 |
| ULBP2 | 2.08 | LIG3 | 0.48 | CDC42EP3 | 2.53 |
| TBL1XR1 | 0.45 | ESCO2 | 2.57 | MXD1 | 2.03 |
| Symbol | Fold-change | Symbol | Fold-change | Symbol | Fold-change |
| KMO | 2.57 | SSH1 | 2.31 | PAK2 | 2.15 |
| ULBP2 | 2.22 | PIK3C3 | 0.49 | CLIC2 | 2.14 |
| PTPRB | 0.45 | SORBS2 | 0.23 | GBA2 | 2.58 |
| WIPI2 | 0.43 | CRLF2 | 2.02 | SLC7A2 | 0.49 |
| CCDC132 | 2.01 | AHNAK2 | 0.49 | JUN | 2.32 |
| INPP4B | 4.16 | UBN2 | 0.48 | LMCD1 | 5.77 |
| PTX3 | 4.92 | FN1 | 2.13 | LOC151760 | 2.56 |
| EFHC2 | 2.2 | FBXO22 | 2.1 | CRISPLD2 | 2.67 |
| AGTR1 | 2.71 | TGFB2 | 2.13 | TNFAIP6 | 2.23 |
| GADD45A | 2.23 | CPS1-IT1 | 0.46 | EXOSC1 | 0.3 |
| PREPL | 2.61 | SHC4 | 2.86 | LMO7 | 0.5 |
| C3ORF52 | 2.2 | CCL5 | 2.35 | TNFAIP6 | 2.2 |
| DOCK10 | 2.54 | HDAC9 | 2.48 | CYP2E1 | 0.47 |
| AFF4 | 2.1 | DIO2 | 2.17 | CHRM3 | 0.36 |
| PPFIBP1 | 2.28 | COL5A2 | 2.16 | CD276 | 2.04 |
| MAP2K6 | 0.44 | LNPEP | 2.17 | COG8 | 0.48 |
| RIBC2 | 0.39 | DGKH | 2.31 | THBS1 | 2.89 |
| CFLAR | 0.43 | CXCL10 | 2.06 | TGFB2 | 2.23 |
| TMEM27 | 2.72 | MMP24-AS1 | 0.4 | IFI44L | 2.28 |
| LOC100132356 | 0.32 | LRRC41 | 2.06 | R3HDM2 | 0.44 |
| PPM1A | 0.45 | MYL9 | 2.06 | TLR4 | 2.44 |
| LINC01588 | 2.02 | OLFML2A | 0.48 | USP7 | 0.37 |
| CBX3 | 0.5 | TMEM79 | 2.14 | CELSR2 | 0.48 |
| SMU1 | 0.39 | IFI27 | 2.66 | FOXP1 | 0.44 |
| TFPI2 | 2.54 | H2AFY | 0.47 | MECP2 | 0.41 |
| Symbol | Fold-change | Symbol | Fold-change | Symbol | Fold-change |
| FAS | 2.1 | COL5A2 | 2.05 | TMEM129 | 0.49 |
| CCSAP | 2.11 | ARHGDIB | 2.32 | LOC101928955 | 2.55 |
| PIK3IP1 | 2.56 | GEMIN8 | 0.39 | RUNDC3B | 2.63 |
| IL13RA2 | 3.47 | TFEC | 7.19 | TRIM24 | 0.27 |
| SF3B2 | 2.01 | PLEKHO1 | 2.91 | PDLIM5 | 2.59 |
| IL1A | 2.51 | DNAJC21 | 2.05 | IL1RN | 2.12 |
| PRDM11 | 0.37 | TMEM64 | 2.13 | WNT2B | 0.22 |
| MFAP2 | 2.07 | SEMA3A | 2.27 | SRSF4 | 0.49 |
| RBM8A | 0.48 | TMEM106B | 0.49 | PPP2R5C | 2.06 |
| YOD1 | 2.17 | JAK2 | 2.39 | MIPEPP3 | 0.49 |
| EEF1A1 | 0.43 | HSPA4 | 2.06 | FCF1 | 2.04 |
| RGS4 | 3.15 | ACCS | 0.49 | ING5 | 0.41 |
| DST | 2.11 | TBC1D17 | 0.42 | PSMG4 | 2.34 |
| PGAP1 | 2.1 | CNOT2 | 2.1 | LOC100505874 | 0.49 |
| SPRED1 | 2.1 | STEAP4 | 2.02 | LOC101929132 | 0.37 |
| KLHL24 | 0.43 | LOC286161 | 0.37 | RPH3A | 2.87 |
| FASTKD2 | 0.43 | DNAJA4 | 2.06 | TNFRSF9 | 2.61 |
| TDO2 | 2.33 | ANKRD36B | 0.48 | PRKCE | 2.02 |
| HMGA2 | 2.66 | CTSS | 2.04 | DDX11L2 | 0.44 |
| SORBS2 | 0.43 | TRPC1 | 0.5 | ZNF397 | 0.45 |
| KLF4 | 2.14 | ABCC3 | 0.41 | GPNMB | 2.41 |
| ODF2L | 0.45 | LOC101928068 | 0.39 | LOC100630923 | 2.3 |
| BTC | 2.2 | MAFB | 2.06 | HIST1H4H | 2.2 |
| EIF3H | 0.36 | USH1C | 0.44 | EMP1 | 2.07 |
| FBXL17 | 0.42 | DESI2 | 2.16 | THBS1 | 2.41 |
| Symbol | Fold-change | Symbol | Fold-change | Symbol | Fold-change |
| THBS1 | 2.4 | GLIPR1 | 2.23 | OCIAD1 | 0.48 |
| DLGAP1-AS2 | 0.47 | PDE1C | 2.59 | GDPGP1 | 0.48 |
| TRIM22 | 2.81 | HOOK3 | 2.08 | APOE | 2.61 |
| ZSCAN20 | 2.98 | BCL2A1 | 3.07 | TM4SF1 | 0.33 |
| RUNDC3B | 2.3 | ACTR2 | 2.41 | LOC728730 | 0.22 |
| SHISA4 | 2.03 | UBASH3B | 2.05 | TAB3 | 2.05 |
| LOC729680 | 0.44 | ATP8A2 | 2.09 | THBS1 | 2.63 |
| LOC100130476 | 3.54 | PACS1 | 2.1 | KLHL24 | 0.39 |
| IQSEC2 | 0.39 | SCARA3 | 2.05 | CXCL11 | 5.37 |
| WDR91 | 0.22 | TAF13 | 2.18 | IL1R2 | 0.49 |
| KANSL1L | 0.43 | LAMA2 | 2.77 | KLHL24 | 0.38 |
| CNTRL | 0.39 | GTF3C4 | 2.45 | H1F0 | 0.49 |
| VAMP2 | 0.29 | LOC102724851 | 0.48 | CCDC15 | 0.42 |
| PLCB4 | 2.07 | SYNCRIP | 0.47 | RARA-AS1 | 0.49 |
| JRK | 0.46 | USH1C | 0.35 | ITGA6 | 2.54 |
| SLIT2 | 2.21 | KLHL41 | 2.29 | BTC | 2.14 |
| KHDRBS1 | 2.34 | ATP6V0D2 | 2.09 |  |  |
| ZBED4 | 0.35 | WDFY3-AS2 | 2.16 |  |  |
| CXCL8 | 2.67 | NFYC-AS1 | 0.49 |  |  |
| INTS4 | 2.42 | ZNF569 | 2.61 |  |  |
| CEP83 | 2.59 | LLPH | 3.33 |  |  |
| CPM | 0.48 | ABCG1 | 0.44 |  |  |
| ICE2 | 2.13 | GOSR2 | 0.41 |  |  |
| DCUN1D1 | 0.45 | HPGD | 2.23 |  |  |
| C11ORF57 | 0.47 | PCNXL4 | 0.31 |  |  |
