# Supplementary material for: Clinical significance of mitofusin-2 and its signaling pathways in hepatocellular carcinoma
Source: World J Surg Oncol. 2016 Jul 7;14:179. doi: 10.1186/s12957-016-0922-5 (PMC4936233; doi:10.1186/s12957-016-0922-5)
Supplement: Additional file 4: Table S3: — Significant enrichment of 93 genes in 18 pathways. (DOC 47 kb) [file 12957_2016_922_MOESM4_ESM.doc]

Supplementary Table 3 Significant enrichment of 93 genes in 18 pathways

| **Pathway** | **Ratio Of Gene In Pathway** | **Number Of Gene In Pathway** | **Gene From Network** | **P-value** | **FDR** | **Gene Set** |
| --- | --- | --- | --- | --- | --- | --- |
| **IL1-mediated signaling events(N)** | 0.0035 | 34 | 5 | 0 | 0.001857 | MAP2K6,IL1A,JUN,IL1R2,IL1RN |
| **Interleukin-1 signaling(R)** | 0.0041 | 40 | 6 | 0 | 0.0003333 | BTRC,MAP2K6,IL1A,IL1R2,IL1RN,TAB3 |
| **Validated transcriptional targets of TAp63 isoforms(N)** | 0.005 | 49 | 5 | 0.0001 | 0.003333 | BTRC,FAS,GADD45A,DST,SP1 |
| **ATF-2 transcription factor network(N)** | 0.0059 | 58 | 5 | 0.0002 | 0.004125 | TGFB2,H2AFY,JUN,GADD45A,CXCL8 |
| **Validated targets of C-MYC transcriptional repression(N)** | 0.006 | 59 | 5 | 0.0002 | 0.004222 | GADD45A,CCL5,CFLAR,SP1,ITGA6 |
| **Beta1 integrin cell surface interactions(N)** | 0.0068 | 66 | 6 | 0 | 0.001889 | THBS1,FN1,SPP1,COL5A2,LAMA2,ITGA6 |
| **ECM-receptor interaction(K)** | 0.0089 | 87 | 6 | 0.0001 | 0.004 | THBS1,FN1,SPP1,COL5A2,LAMA2,ITGA6 |
| **Rheumatoid arthritis(K)** | 0.0092 | 90 | 6 | 0.0002 | 0.004133 | TLR4,TGFB2,IL1A,JUN,CXCL8,CCL5 |
| **NF-kappa B signaling pathway(K)** | 0.0093 | 91 | 7 | 0 | 0.001625 | TLR4,BCL2A1,UBE2I,CXCL8,CFLAR,TAB3,DDX58 |
| **Chagas disease (American trypanosomiasis)(K)** | 0.0107 | 104 | 8 | 0 | 0.0015 | TLR4,TGFB2,PLCB4,FAS,JUN,CXCL8,CCL5,CFLAR |
| **Toll-like receptor signaling pathway(K)** | 0.0109 | 106 | 8 | 0 | 0.0012 | TLR4,CXCL11,CXCL10,MAP2K6,JUN,CXCL8,CCL5,SPP1 |
| **Amoebiasis(K)** | 0.0112 | 109 | 8 | 0 | 0.001167 | TLR4,TGFB2,PLCB4,IL1R2,CXCL8,FN1,COL5A2,LAMA2 |
| **TNF signaling pathway(K)** | 0.0113 | 110 | 7 | 0.0001 | 0.0032 | CXCL10,FAS,MAP2K6,JUN,CCL5,CFLAR,TAB3 |
| **Direct p53 effectors(N)** | 0.0135 | 132 | 7 | 0.0002 | 0.004294 | PTEN,FAS,BCL2A1,JUN,GADD45A,SPP1,SP1 |
| **Influenza A(K)** | 0.018 | 176 | 11 | 0 | <5.000e-04 | TLR4,CXCL10,FAS,DNAJC3,MAP2K6,IL1A,JUN,CXCL8,CCL5,DDX58,JAK2 |
| **Focal adhesion(K)** | 0.0212 | 207 | 12 | 0 | <1.000e-03 | PTEN,PAK2,SHC4,JUN,MYL9,IGF1R,THBS1,FN1,SPP1,COL5A2,LAMA2,ITGA6 |
| **Cytokine-cytokine receptor interaction(K)** | 0.0272 | 265 | 10 | 0.0001 | 0.004308 | CXCL11,CXCL10,TGFB2,FAS,IL1A,IL1R2,CRLF2,CXCL8,IL7R,CCL5 |
| **PI3K-Akt signaling pathway(K)** | 0.0356 | 347 | 12 | 0.0001 | 0.002909 | PPP2R5C,TLR4,PTEN,IL7R,IGF1R,THBS1,FN1,SPP1,COL5A2,LAMA2,ITGA6,JAK2 |
