# Supplementary figures and images for: Clinical significance of mitofusin-2 and its signaling pathways in hepatocellular carcinoma
Source: World J Surg Oncol. 2016 Jul 7;14:179. doi: 10.1186/s12957-016-0922-5 (PMC4936233; doi:10.1186/s12957-016-0922-5)

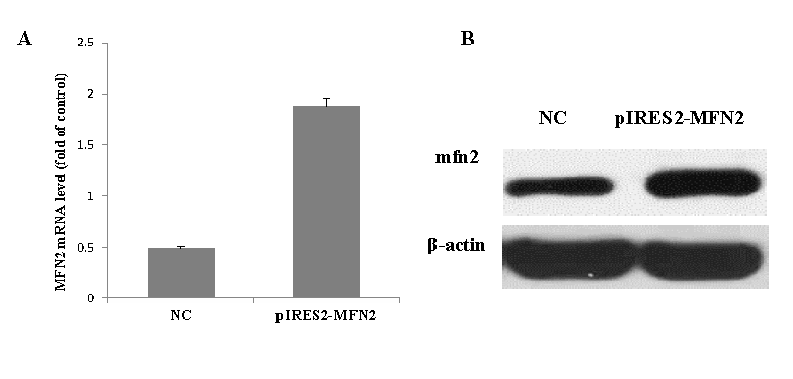

Supplement: Additional file 5: Figure S2: — Efficiency of transfection evaluation by qPCR and western blot analysis in HepG2 cells. (A) MFN2 mRNA was significantly upregulated by plasmid-MFN2. Standard deviation (SD) was used as error bar. (B) MFN2 protein was also upregulated by plasmid-MFN2. (TIF 46 kb) [file 12957_2016_922_MOESM5_ESM.tif]
